# Supplementary figures and images for: Sensing and signaling of immunogenic extracellular RNAs restrain group 2 innate lymphoid cell-driven acute lung inflammation and airway hyperresponsiveness
Source: PLoS One. 2020 Jul 30;15(7):e0236744. doi: 10.1371/journal.pone.0236744 (PMC7392318; doi:10.1371/journal.pone.0236744)

## Slide 1
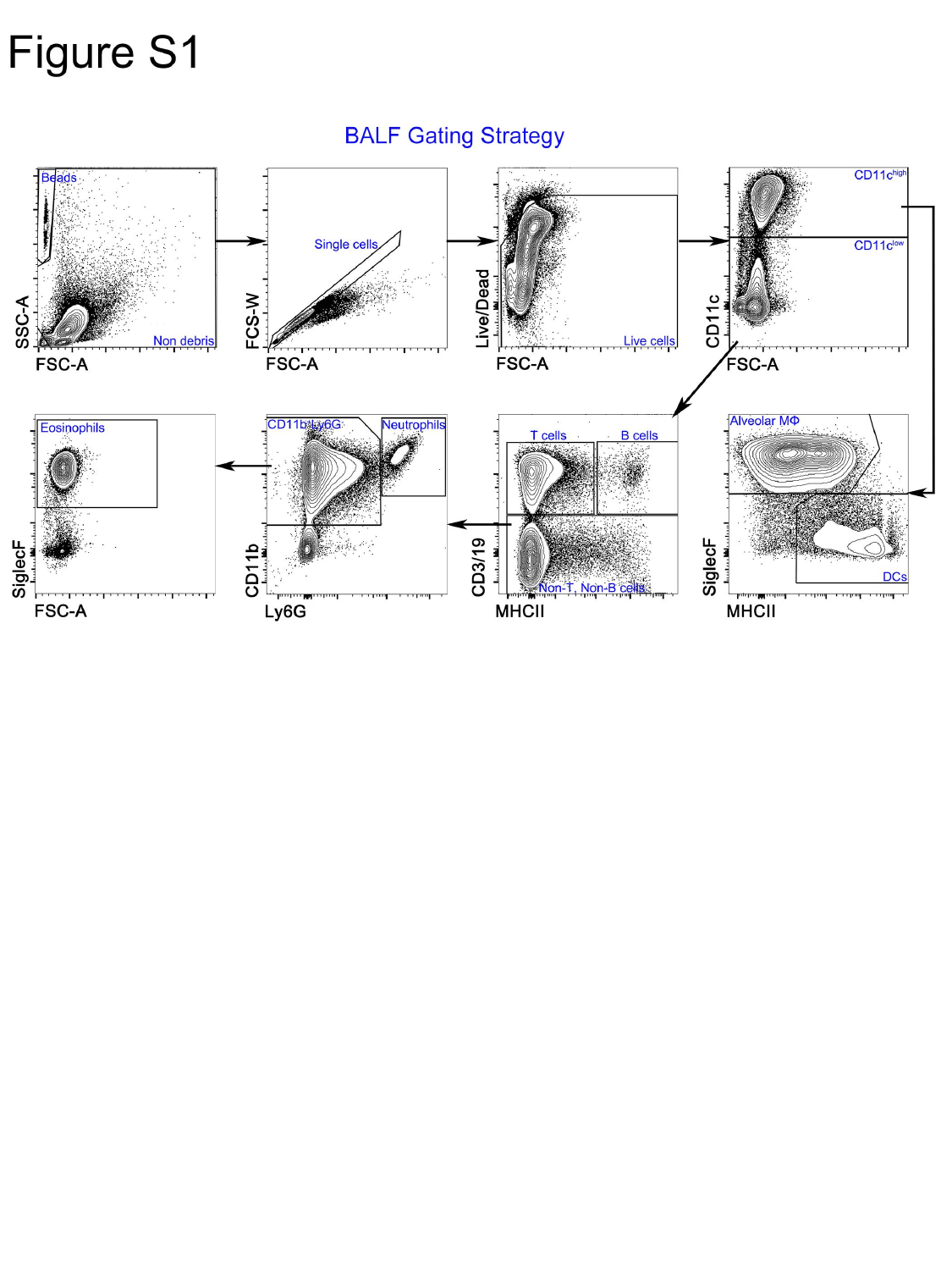

Supplement: S1 Fig — Cells recovered from BALF were stained for cell surface markers as indicated. The absolute numbers were calculated based on reference beads (top panel). Formula (Number of cells per mL): (Total Beads/# events of Beads) x (# event of Sample)/Volume of Tested Sample. (Related to Figs 1C, 2C, 3C, 5A, 6A, 7A, 8A, 9A and S3A). (PPTX) [file pone.0236744.s001.pptx]

## Slide 1
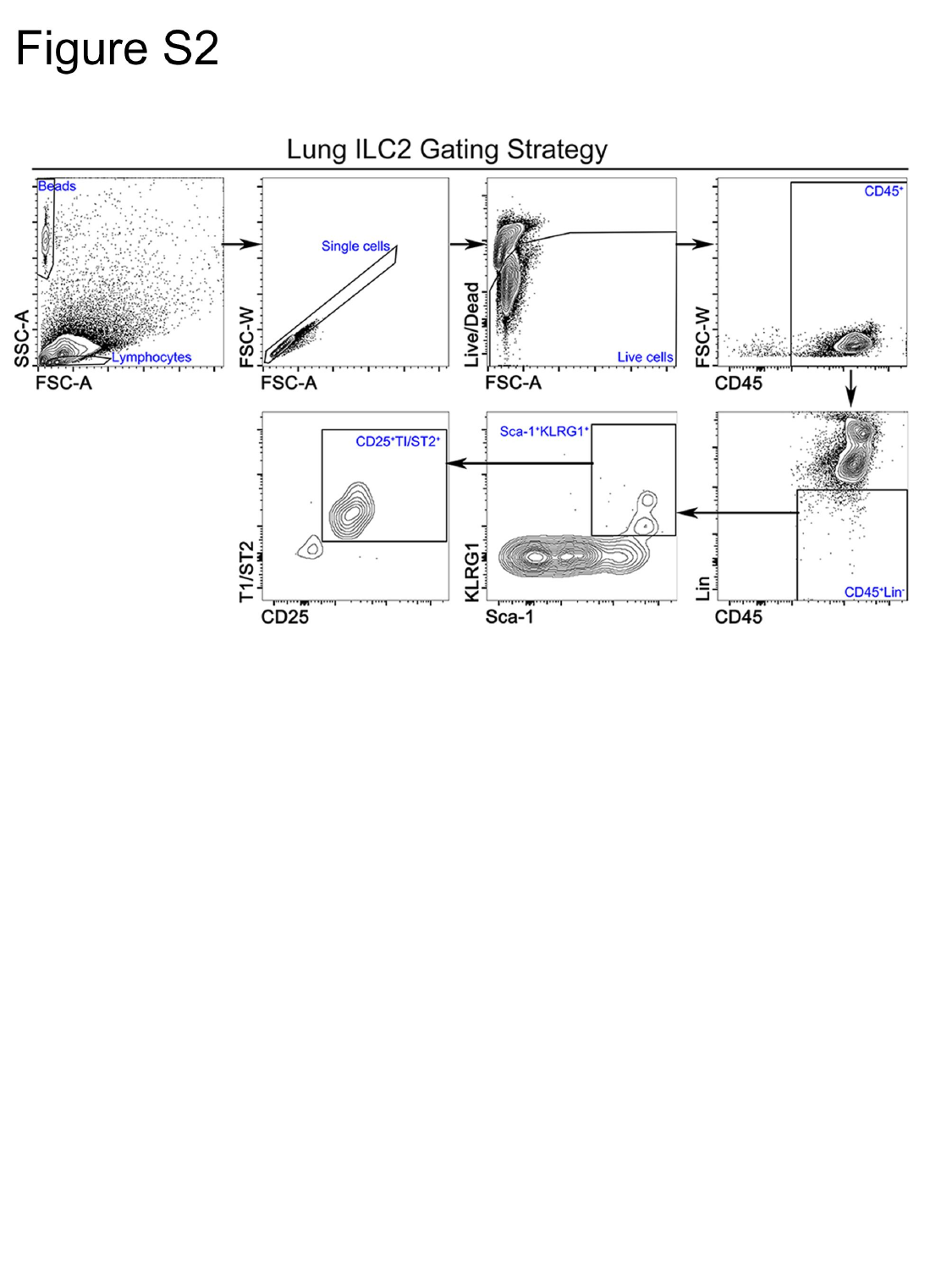

Supplement: S2 Fig — Cells recovered from total lung homogenates were stained for cell surface markers as indicated. The absolute numbers were calculated based on reference beads (top panel). Formula (Number of cells per mL): (Total Beads/# events of Beads) x (# event of Sample)/Volume of Tested Sample. (Related to Figs 1E, 4B–4D, 5C, 6C, 7C, 8C, 9C and S5C). (PPTX) [file pone.0236744.s002.pptx]

## Slide 1
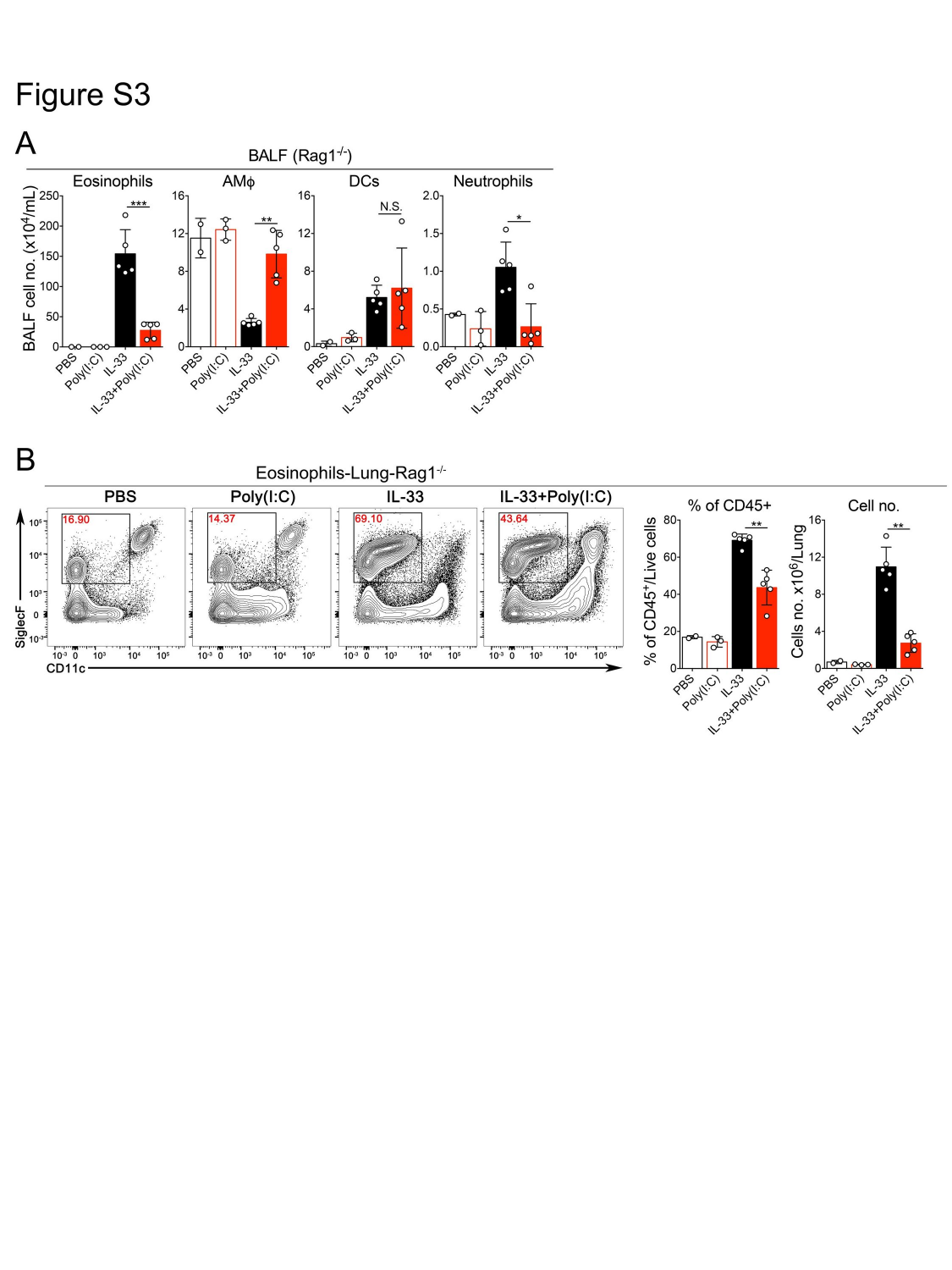

Supplement: S3 Fig — A. Four groups of Rag1-/- mice were treated with PBS, Poly(I:C), IL-33 or IL-33+Poly(I:C) as indicated. BALF was collected and analyzed for differential immune cell types. B. Administration of Poly(I:C) decreased the percentage and number of eosinophils in lungs after exposure to IL-33. (n = 2–5 per group as indicated with open circles, P value was determined using Mann-Whitney test, * p < 0.05, ** p < 0.01, *** p < 0.001). (PPTX) [file pone.0236744.s003.pptx]

## Slide 1
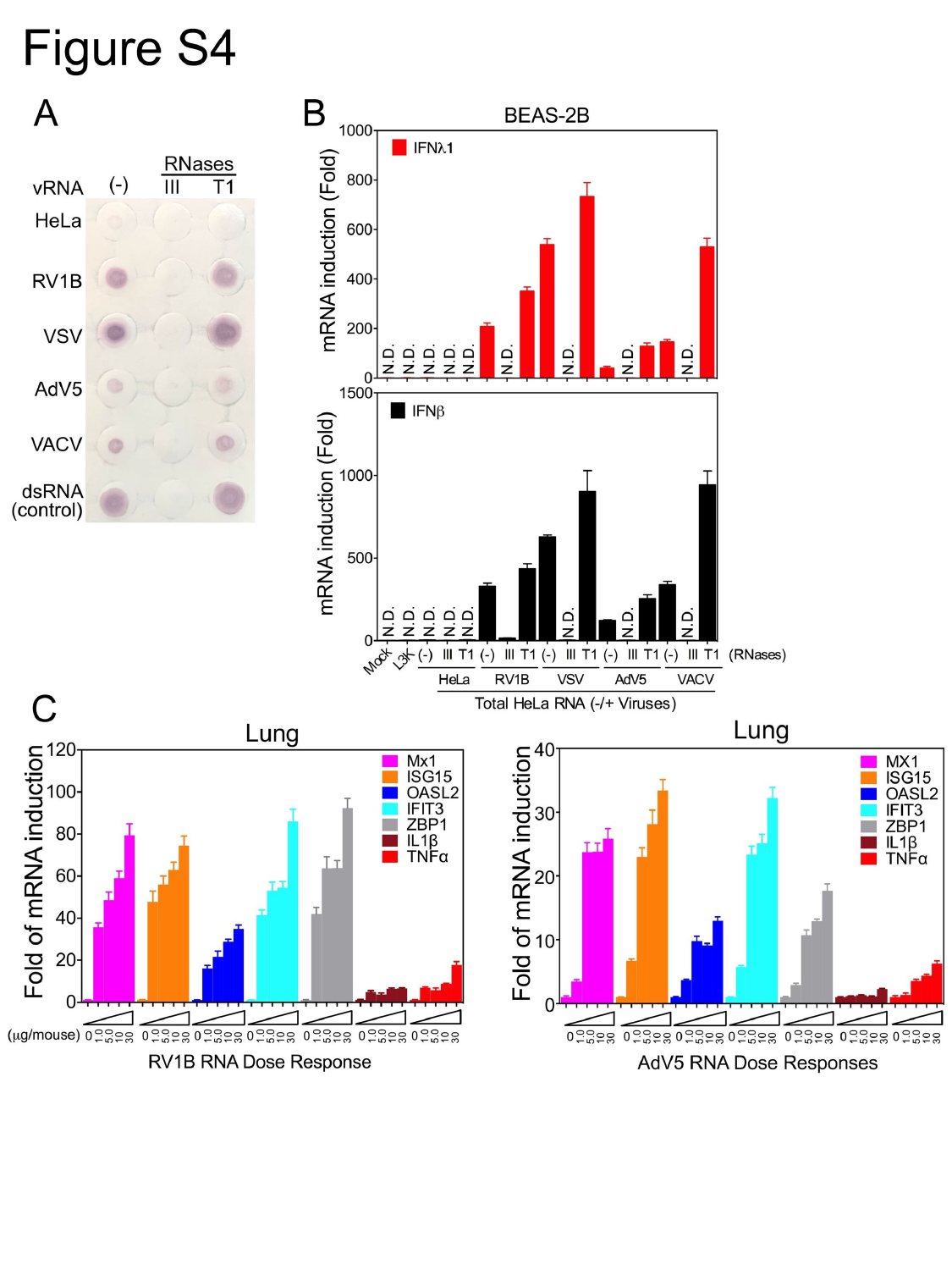

Supplement: S4 Fig — . A. Detection of the double-stranded structure in total RNA isolated from virus-infected HeLa cells by a dsRNA-specific antibody J2 with Dot Blot. B. Induction of IFNs by RNAs produced by HeLa cells infected with both RNA- and DNA-viruses. BEAS-2B cells were transfected with total RNAs (1.0 μg /ml per 0.2x10^6^ cells) of HeLa cells infected with viruses for 16–18 hours. Total RNAs were also treated with or without RNases III or T1 as indicated. C. Transcriptional induction of gene expressions by RV1B RNA in mouse lungs. Wild type mice were exposed via the intratracheal route to increased amounts of RV1B RNA as indicated. After 16-18h, total RNA isolated from mouse lungs was subjected to RT-qPCR analysis. (Related to Figs 8 & 9). N.D., not detected. (PPTX) [file pone.0236744.s004.pptx]

## Slide 1
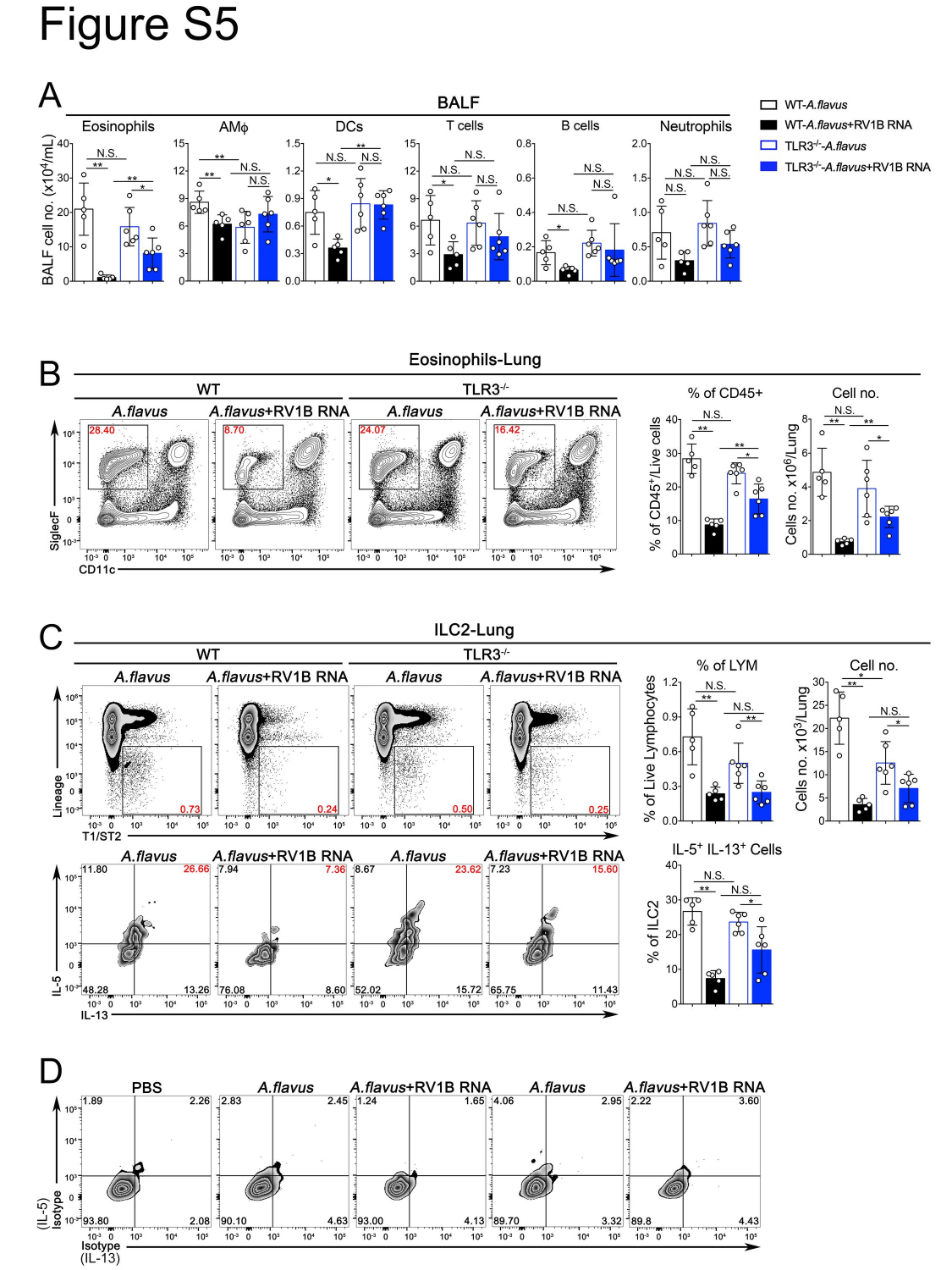

Supplement: S5 Fig — A. Groups of WT and TLR3-/- mice as indicated were treated with A. flavus or A. flavus+RV1B RNA. BALF was collected and analyzed for differential immune cell types. B. The percentage and number of eosinophils cells in lungs of WT and TLR3-/- mice were analyzed. C. The percentage and number of ILC2 cells and percentage of IL5+/IL13+-double positive ILC2 cells in lungs of WT and TLR3-/- mice were analyzed. (n = 5–6 per group as indicated with open circles, P value was determined using Mann-Whitney test, P value ≥0.05 was not considered statistically significant [N.S.]). * p < 0.05, ** p < 0.01). D. Cells were stained with the isotype antibodies corresponding to IL-5 and IL-13. (PPTX) [file pone.0236744.s005.pptx]
